# Lipid droplets-related Perilipin-3: potential immune checkpoint and oncogene in oral squamous cell carcinoma

**DOI:** 10.1007/s00262-024-03659-9

**Published:** 2024-03-30

**Authors:** Yijia He, Lingyun Liu, Yuexin Dong, Xiaoxin Zhang, Yuxian Song, Yue Jing, Yanhong Ni, Yi Wang, Zhiyong Wang, Liang Ding

**Affiliations:** 1 Central Laboratory of Stomatology, Nanjing Stomatological Hospital, Affiliated Hospital of Medical School, Nanjing University, Nanjing, China; 2 Department of Oral and Maxillofacial Surgery, Nanjing Stomatological Hospital, Affiliated Hospital of Medical School, Nanjing University, Nanjing, China

**Keywords:** OSCC, Lipid droplets, Perilipin-3, PD-L1, B7-H2

## Abstract

**Background:**

Lipid droplets (LDs) as major lipid storage organelles are recently reported to be innate immune hubs. Perilipin-3 (PLIN3) is indispensable for the formation and accumulation of LDs. Since cancer patients show dysregulated lipid metabolism, we aimed to elaborate the role of LDs-related PLIN3 in oral squamous cell carcinoma (OSCC).

**Methods:**

PLIN3 expression patterns (*n* = 87), its immune-related landscape (*n* = 74) and association with B7-H2 (*n* = 51) were assessed by immunohistochemistry and flow cytometry. Real-time PCR, Western blot, Oil Red O assay, immunofluorescence, migration assay, spheroid-forming assay and flow cytometry were performed for function analysis.

**Results:**

Spotted LDs-like PLIN3 staining was dominantly enriched in tumor cells than other cell types. PLIN3^high^ tumor showed high proliferation index with metastasis potential, accompanied with less CD3^+^CD8^+^ T cells in peripheral blood and in situ tissue, conferring immunosuppressive microenvironment and shorter postoperative survival. Consistently, PLIN3 knockdown in tumor cells not only reduced LD deposits and tumor migration, but benefited for CD8^+^ T cells activation in co-culture system with decreased B7-H2. An OSCC subpopulation harbored PLIN3^high^B7-H2^high^ tumor showed more T cells exhaustion, rendering higher risk of cancer-related death (95% CI 1.285–6.851).

**Conclusions:**

LDs marker PLIN3 may be a novel immunotherapeutic target in OSCC.

## Introduction

Oral squamous cell carcinoma (OSCC) is the most common histological subtype in oral cancer, and often allied with relapse [1]. Despite recent advances in traditional treatment such as surgery, chemotherapy, radiotherapy or a combination of these modalities [2], unfortunately, the survival of OSCC patients has remained relatively unchanged for the last several decades and the main reason for treatment failure is locoregional recurrence [3]. Therefore, discovering new mechanisms and biomarkers to aid in the diagnosis and treatment of OSCC is needed to achieve better patient outcomes. Recently, we previously found that stromal IL-33/ST2 signaling directly or indirectly enhanced PD-L1 (B7-H1)-mediated immune escape [4, 5] and also revealed that OXTR^+^ stromal fibroblasts induced high tumor invasion potential and postoperative recurrence in partial OSCC subgroup [6].

It appears that metabolic disruptions in cancer cells may not only be indicative of cancer, but could also be a contributing factor to the development of tumors, such as alterations in glucose, glutamine, and lipid metabolism [7]. Cancer cells take advantage of lipid to generate energy, form biofilms, and generate signaling molecules necessary for growth, survival, invasion, and metastasis [8]. Lipid droplets (LDs) participate in lipid metabolism. Structurally, LDs consist of a neutral lipid core surrounded by a phospholipid monolayer that contains a diverse array of embedded proteins [9], including perilipins (PLIN), a family of proteins that involved in the synthesis of neutral lipids [10]. Importantly, among major members of PLIN family, PLIN2 and PLIN3 consistently play active roles in intracellular lipid storage, nascent LD biogenesis, and the lipolysis of LDs, while other PLIN isoforms remained unaltered [11–15]. However, for many years, the study of PLIN2 and PLIN3 in cancer was restricted to descriptions in different tumors. Some studies reported that in cutaneous melanomas, pancreatic ductal adenocarcinoma, breast cancer, and lung adenocarcinomas, the overexpression of PLIN2 was strongly associated with adverse outcome [16–19], while in prostate cancer, renal clear cell carcinoma, cervical cancer, and liver cancer, PLIN3 promoted tumor progression [20–24]. Although PLIN2 and PLIN3 have clinical utility in specific tumors, research to prove their validity in OSCC was lacking.

Abnormal lipid metabolism can enable tumor cells to establish an immunosuppressive network [25] and multiple immune proteins nucleated around PLIN2 in response to lipopolysaccharide [26]. Similarly, we discovered that CD68^+^ PLIN2^+^ tumor-associated macrophages induced immune suppression, accompanied by high levels of immune checkpoint molecules [27]. Previous studies found that PLIN3 can participate in the transformation of macrophages and promote the expression of Toll-like receptor 9 to activate the immune response [28, 29]. However, the potential interactions between PLIN3 and other main components of the tumor microenvironment (TME), especially immunocytes in OSCC, are yet to be elucidated.

In this work, immunohistochemistry (IHC) staining of OSCC and single-cell RNA sequencing (scRNA-seq) in head and neck squamous cell cancer (HNSCC) tissue show the location of PLIN3 in tumor. Further, we used IHC serial sections and peripheral blood samples to analyze the intricate crosstalk with PLIN3 and immunocytes in OSCC. Furthermore, in vitro and in vivo functional assays including OSCC/T cell co-culture system were performed to validate the malignant function and the underlying mechanism of PLIN3, providing new clues for guiding clinical prevention and treatment of OSCC.

## Materials and methods

### Patients and samples

The studies involving human participants were reviewed and approved by the Ethics Committee of Nanjing Stomatology Hospital (2019NL-009(KS)). OSCC tumors were obtained from 87 patients from 2014 to 2015 in Nanjing Stomatological Hospital. Informed consent was provided by the patients for the use of their tissues and data. All cases were reviewed by experienced pathologists. Paraffin-embedded OSCC tissue slices were obtained from the pathology department and used for immunohistochemistry study. Seventy-four blood samples from OSCC patients were obtained for flow cytometry assay before any related treatments. Additional 51 OSCC samples were used to determine the correlation between PLIN3 and B7-H2. The 51 samples tested by flow cytometry and 87 samples tested by IHC were all primary OSCC patients who had not received preoperative radiotherapy or chemotherapy.

### Immunohistochemistry and quantification

The protocol of immunohistochemistry of formalin-fixed paraffin-embedded sections was performed as previously described [30]. The primary antibodies include PLIN3 (diluted 1:500, Abcam, ab224344), B7-H2 (diluted 1:400, Abcam, ab257321), Ki-67 (diluted 1:200, ab16667; Abcam), CD4 (ZSGB-BIO, ZM-0418), CD8 (ZSGB-BIO, ZA-0508), CD19 (ZSGB-BIO, ZM-0038), CD56 (ZSGB-BIO, ZM-0057), and CD68 (ZSGB-BIO, ZM-0464). The CD4, CD8, CD19, CD56, and CD68 antibody working solution did not need to be diluted and was used directly by dropwise addition. The assessment of antigen expression was based on the intensity of the stain and the proportion of cells that tested positive. The percentage of stained cells was defined as 0 = 0–5%; 1 = 6%–25%; 2 = 26%–50%;3 = 51%–75%; and 4 = 76–100%. Staining intensity was defined as follows: 0 = negative staining; 0 = no staining; 1 = light yellow; 2 = brownish yellow; 3 = dark brownish yellow. The IHC score was calculated by multiplying the two scores as mentioned above. The expression levels of PLIN3, B7-H2, Ki-67, and immunocyte molecules were defined as “low” when it is lower than the median value and as “high” when it is equal to or greater than the median. Two pathologists conducted all scorings without any knowledge of the patients' clinical characteristics or outcome. Define polygonal or long spindle-shaped cells as FLCs with ovoid nuclei and large, distinct nucleoli, whereas TILs have small cytoplasmic bodies and a smaller nucleoplasmic ratio and are often more regular.

### Database analysis

We used the GEO (GSE103322, GSE164690), TISCH (http://tisch.comp-genomics.org/) to analyze the RNA expression of *PLIN3* and used the Human Protein Atlas (https://www.proteinatlas.org/) database to analyze the protein levels of PLIN3 in different cell types of HNSCC and tongue tissues. We used cBioportal (https://www.cbioportal.org/) and TIMER (http://timer.cistrome.org/) database to analyze the correlation between RNA expression of *PLIN3* and specific immune cell subsets markers in HNSCC.

### Cell culture and reagents

OSCC cell lines (HN6, Cal27, and Cal33) and 293 T cells were obtained from the National Collection of Authenticated Cell Cultures and identified by Short Tandem Repeat. HN6, Cal27, Cal33, and 293 T cells were cultured in DMEM medium (Invitrogen) supplemented with 10% Fetal bovine serum (FBS) (Gibco) at 37 °C in 5% CO_2_. siRNA and lentiviral plasmid vectors of PLIN3 were purchased from RiboBio and OBIO, respectively. Oleic acid (Merck, O1257) was diluted at a ratio of 1:500 and added to cells for 12 h. All steps were executed according to the manufacturer's instructed protocol.

### Cell transfection

Cells were grown on 12/24 wells plate to 50% confluence and transfected the siRNA of PLIN3 using Lipofectamine 3000 (Invitrogen) according to the manufacturer’s instructions. Cells were harvested after 24 h or 48 h for qPCR, Western blot analysis, and RNA sequence (Shanghai OE Biotech. Co., Ltd).

### Lentivirus vectors

As for the stable transfection of PLIN3, 293 T cells were cultured in 10-cm dish and transfected with Lentiviral plasmid vector using PolyJetTM DNA in vitro transfection reagents (SignaGen, America), the virus supernatant above 293 T cells was extracted and cultured with tumor cells. The GFP-labeled Lentivirus vector expressing sh-PLIN3 (Lv-ShPLIN3) and overexpression vector containing PLIN3 (Lv-OE-PLIN3) were used, with Lv-ctrl as the matched controls (OBiO Technology Co. Ltd., Shanghai, China).

### RNA analysis

RNA was obtained by using AG SteadyPure RNA Extraction Kit following the manufacturer’s procedure and then reversed into cDNA using 5X Evo M-MLVRT Master Mix (Accurate Biotechnology, AG11706). The relevant expression of the genes was determined via 2X SYBR Green Pro Taq HS Premix (Accurate Biotechnology, AG11718).

### Western blot analysis

The protocol of WB was performed as previously described [5]. Protein samples were isolated by 4–20% SDS-PAGE (smart-Lifesciences). The primary antibodies include PLIN3 (1:1000, Abcam, ab224344), B7-H2(1:1000, Abcam, ab257321), PD-L1(1:1000, CST, #13684), and ACTIN (1:2000, Servicebio, GB12001).

### RNA sequencing

The RNA sequencing was performed by OEbiotech. Briefly, the collected cells were subjected to RNA isolation using SteadyPure RNA Extraction Kit (AG) according to the instructions. In the constructed RNA library, rRNA was removed using the TruSeq Stranded Total RNA with Ribo-Zero Gold kit and cDNA was reverse transcribed. Purified DNA was amplified by QPCR. The libraries were quantified and sequenced (Illumina, San Diego, CA). Differentially expressed mRNAs were further analyzed according to *p *values < 0.05.

### Pathway enrichment analysis

GSEA includes GO enrichment and KEGG pathway assays, employed to unveil the fundamental biological process, cellular composition, and functional molecules. Each gene set was assigned a distinct gene set based on a 'hallmark'.

### Oil Red O staining assay

The working Oil Red O solution was obtained by diluting saturated Oil Red O solution (Servicebio technology) at 6:4 with distilled water. The cells which were cultured in 6-well plate were washed twice using PBS and fixed in 4% paraformaldehyde for 10 min before staining, and then the cells were incubated in 60% isopropanol for 15 s and incubated in Oil Red O staining solution avoiding light for 10 min, and finally, washed twice, photographed and counted. Using image J to measure the length of LDs. Oil Red O staining of frozen sections was also carried out according to the instructions [21].

### Immunofluorescence

Cultured cells were fixed in 4% paraformaldehyde solution on the 6-well plates and washed in 1xPBS prior to staining with 10 μg/ml BODIPY 493/503 (Invitrogen, D3922) for 10 min. Samples were then washed with PBS twice. Similarly, cells were incubated with 5 μg/ml Nile red solution (Invitrogen, N1142) for 15 min at room temperature to stain neutral lipids, and the images were visualized by immunofluorescence microscopy.

### Wound healing assay

The constructed stable-transformation cell lines and control cells were seeded in 6-well plates until 100% fusion. The wounds were scored with a micropipette tip and then washed with PBS and cultured in serum-free DMEM medium. The same area of the wound was photographed at 0 and 24 h to analyze the wound closure of the cells. ImageJ software was used to measure the trace widths of different treatment groups for analysis.

### Transwell assay

To assess cell migration capacity, transwell assays were done as described previously [21]. In brief, 2 × 10^4^ cells were seeded in the top chamber of the insert (200 μL/well) with serum-free medium, followed by adding media containing 10% FBS in the lower chamber as a chemoattractant (600 μL/well). Five fields were randomly chosen, and the migration ability of tumor cells was assessed by ImageJ software.

### 3D spheroid models

To assess the proliferative capacity of tumor cells, we used 3D spheroid models. Spheroid formation was achieved by using ultra-low attachment (ULA) culture plates (96-well spheroid microplates with ULA surface). HN6 cells were grown in DMEM supplemented with 10% FBS and 1% penicillin–streptomycin. A total of 8000 cells were seeded per well and cultured in an ultra-low adhesive environment in a humidified 5% CO_2_ atmosphere at 37 °C, and the medium was refreshed once a day. The diameter of tumor spheres was observed daily for the following eight days.

### Preparation of human peripheral blood mononuclear cells (PBMCs)

PBMC was prepared as previously reported [5]. All study participants provided informed consent and approved by the ethical committee of Nanjing Stomatology Hospital, affiliated Hospital of Medical School, Nanjing University, Nanjing, China.

### Co-culture experiment of PBMC and tumor cells

The experimental group and the control group of tumor cells were plated in 12-well plates, and the count of cells in each well was 1 × 10^5^. According to the ratio of PBMC: tumor cells = 5:1, PBMC was added into the 12-well plates and cultured for 48 h, and then flow cytometry was performed.

### Flow cytometry assay

The PBMC samples were collected from patient's peripheral blood. Percentage and absolute numbers of lymphocytes were assessed by flow cytometry. Cells were collected and then washed and suspended in 200μL PBS. Cells were stained with CD45-PerCP, CD3-FITC, CD4-APC, CD8-PE, CD19-APC, CD16-PE, CD56-PE (BD Multitest™) for 30 min to enumerate the CD3^+^CD4^+^ T cells, CD3^+^CD8^+^ T cells, CD3^−^CD19^+^ B cells, and CD3^−^CD16^+^CD56^+^ NK cells, respectively. Flow cytometry was finally performed using FACSCalibur instrument and analyzed using FlowJo software, and the graphs were generated by GraphPad Prism. For the cell subtypes of PBMC analysis, details of this protocol were the same as the previous description [31].

### Statistics

SPSS 18.0 and GraphPad Prism 8.0 software packages were used for data analysis and graphic processing. Pearson's chi-square test and Fisher's exact test were used to compare clinicopathological characteristics. Two-tailed Student's t test was used to compare the two groups of OSCC patients. Survival analysis was performed using the Kaplan–Meyer test and log-rank test. Statistical significance was defined as confidence intervals greater than or equal to 95% or *P* < 0.05. Experiments shown are representative of three different replicates.

## Results

### PLIN3^high^ tumors show high proliferation index with metastasis potential

To unveil the spatial distribution of PLIN3 in vitro, immunohistochemical (IHC) staining was performed in a cohort of OSCC patients (*n* = 87). Fifty-five patients were male, and 32 patients were female. Median patient age was 62 years. The site of occurrence was predominantly the tongue in 87 patients, accounting for 34.5% of all sites. Median follow-up was 30 months for overall survival time (OS), 26 for metastasis-free survival time (MFS), and 21 months for disease-free survival time (DFS). Most patients (*n* = 58) were in advanced stages of OSCC (cTNM III or IV), and 23 patients exhibited postoperative distant metastasis.

**Fig. 1:**
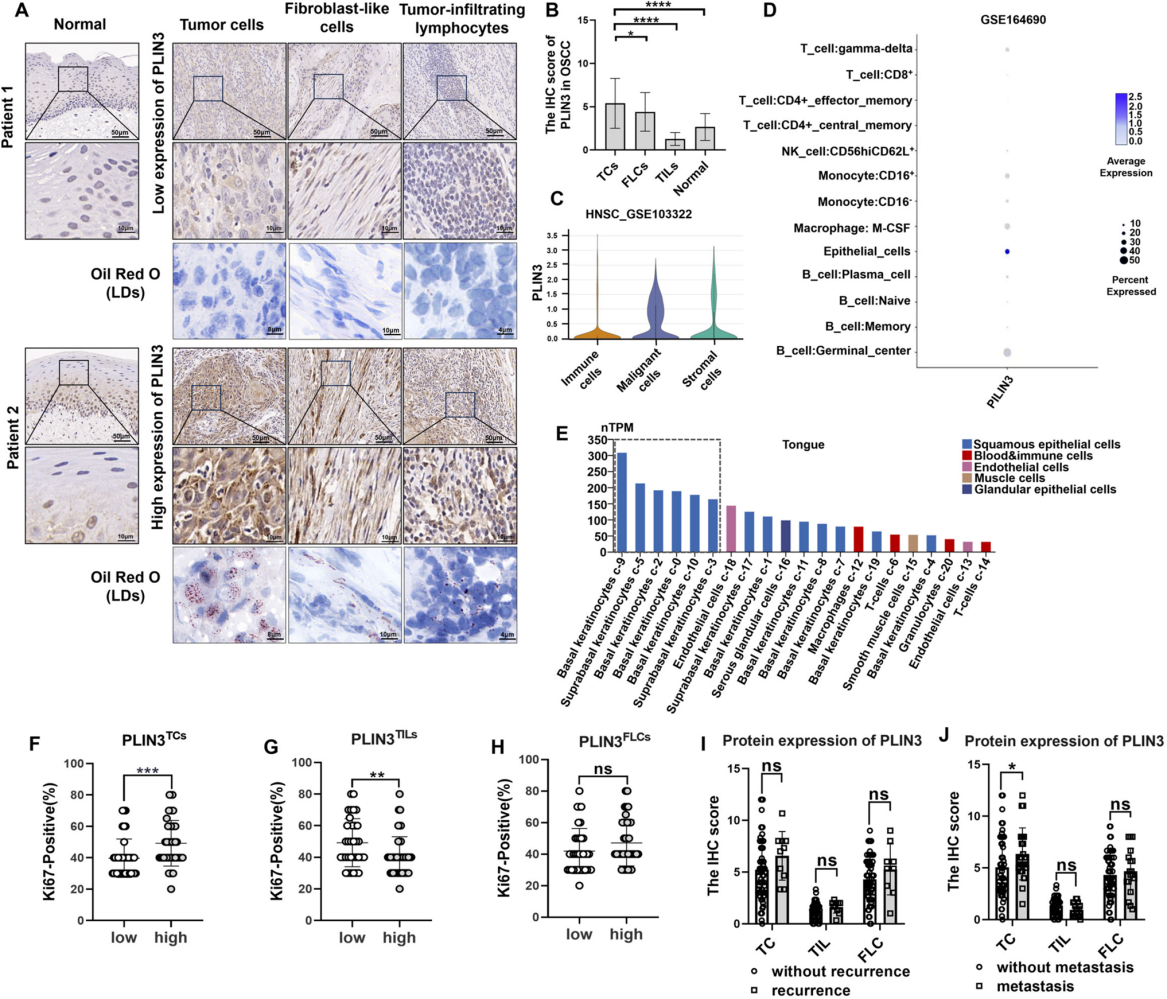
Expression pattern of spotted LDs-like PLIN3 and association with proliferation index and postoperative status. **a** Representative image of IHC (the bar above is 50 µm and below is 10 µm) and Oil Red O (Scale bars for TCs, FLCs, and TILs indicate 8, 10, and 4 µm, respectively) for low and high expression of PLIN3 in normal epithelial, TCs, FLCs, and TILs in OSCC. **b** The IHC score of PLIN3 in normal, TCs, FLCs, and TILs from OSCC patients (*n* = 87). **c, d** Expression of PLIN3 in HNSCC was analyzed by single-cell RNA sequencing (GSE103322, GSE164690). **e** Single-cell RNA-seq results of PLIN3 in tongue tissues (https://www.proteinatlas.org). **f–h** Correlation between Ki-67 staining and PLIN3 in TCs, TILs and FLCs. **i, j** The effect of PLIN3 on postoperative recurrence and metastasis status in OSCC patients were shown by two-way ANOVA (mixed model). Results are shown as mean ± SEM. *p* = two-tailed t test

Histological sections showed that spotted lipid droplets-like PLIN3 staining was dominantly enriched in tumor cells (TCs) than other cell types including fibroblast-like cells (FLCs) and tumor-infiltrating lymphocytes (TILs) (Fig. 1a, b). Similarly, single-cell RNA-seq data revealed that in head and neck squamous cell cancer (HNSCC), PLIN3 was enriched in epithelial cells (Fig. 1c, d) (GSE103322, GSE164690), and a similar expression pattern was also exhibited in tongue tissues (Fig. 1e) (https://www.proteinatlas.org). Interestingly, OSCC patients with PLIN3^high^ tumor cells exhibited a higher Ki-67 positivity (Fig. 1f) contrary to immunocytes (Fig. 1G), and we speculate that PLIN3 was as important as LDs for the growth of tumor cells, but also indispensable for immune cells in the activation of CD8^+^ T lymphocytes [32, 33]. There was no significant link between PLIN3 and Ki-67 in OSCC fibroblasts (Fig. 1h).

Correlation between protein expression levels of PLIN3 and clinicopathological variables was evaluated in patients with OSCC, such as postoperative recurrence, distant metastasis, sex, age of patients, as well as tumor node metastasis (TNM) stage, T stage, N stage, and differentiation. Our data showed that patients with increased PLIN3^TCs^, instead of PLIN3^TILs^ and PLIN3^FLCs^, had a higher risk of postoperative distant metastasis, but the recurrence incidence was comparable in total OSCC cohort (Fig. 1i, j). Notably, we also observed that patients with PLIN3^high^ tumor cells were strongly correlated with poor differentiation and high PLIN3^+^ fibroblasts showed advanced T stage (Table 1), suggesting that PLIN3 in OSCC might promote malignant progression.

**Table 1:** Association between PLIN3 protein expression and clinicopathological characteristics in OSCC patients

Characteristics	N	PLIN3 protein expression in TCs		c^2^	*P*	N	PLIN3 protein expression in FLCs		c^2^	*P*	N	PLIN3 protein expression in TILs		c^2^	*P*
		Low	High				Low	High				Low	High		
*Sex*															
Male	55	25	30	0.024	0.877	47	27	20	3.491	0.062	48	22	26	0.461	0.497
Female	32	14	18			26	9	17			29	11	18		
*Age (years)*															
< 60	32	19	13	4.331	0.037	30	14	16	0.143	0.705	31	12	19	0.364	0.546
≥ 60	55	20	35			43	22	21			46	21	25		
*TNM*															
I–II	30	16	14	1.339	0.247	24	15	9	2.487	0.115	27	9	18	1.540	0.215
III–IV	57	23	34			49	21	28			50	24	26		
*T stage*															
1–2	63	29	34	0.134	0.714	51	30	21	6.121	0.013*	55	22	33	0.642	0.423
3–4	24	10	14			22	6	16			22	11	11		
*N stage*															
No	39	17	22	0.044	0.834	32	16	16	0.011	0.918	35	12	23	1.925	0.165
Yes	48	22	26			41	20	21			42	21	21		
*Differentiation*															
Well	17	12	5	5.669	0.017*	14	9	5	1.553	0.213	15	5	10	0.690	0.406
Moderate to poor	70	27	43			59	27	32			62	28	34		
Total	87					73					77				

*PLIN3* Perilipin 3, *OSCC *oral squamous cell carcinoma, *TCs *tumor cells, *FLCs *fibroblast-like cells, *TILs *tumor-infiltrating lymphocytes

*Represented that differences were considered statistically significant with *P* < 0.05

### PLIN3^high^ tumors harbor an immunosuppressive microenvironment in situ

**Fig. 2:**
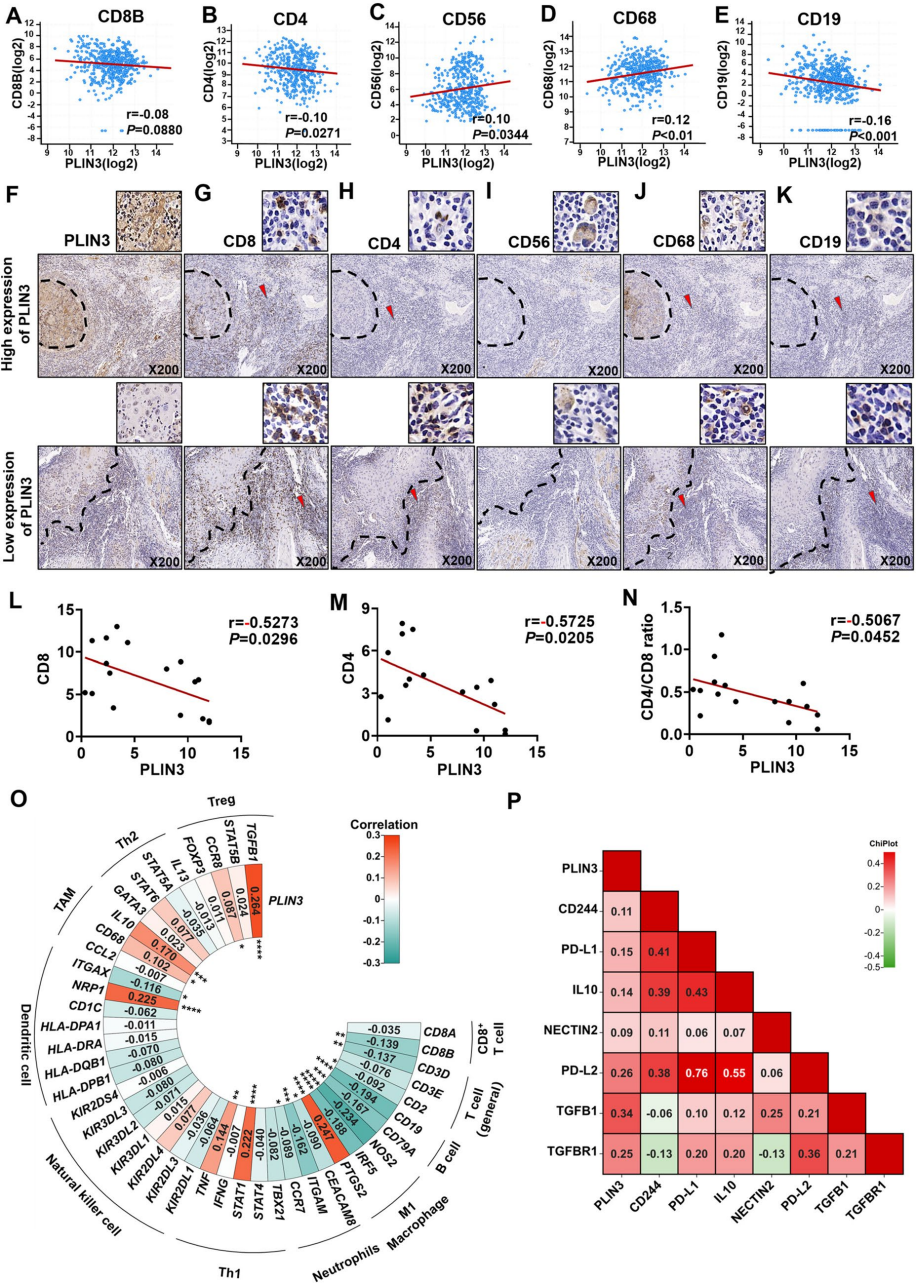
PLIN3 is associated with the immunosuppressive microenvironment of tumor tissue in situ. **a–e** Correlation between *PLIN3* RNA expression and *CD8B*, *CD4*, *CD56*, *CD68,* as well as *CD19* in HNSCC from cBioportal database. **f–k** Relationship between PLIN3 expression and CD8^+^ T cell, CD4^+^ T cell, CD56^+^ NK cell, CD68^+^ TAM, and CD19^+^ B cell infiltration in serial sections of OSCC followed by IHC (*n* = 10). **l–n** Quantitative statistics on the correlation between PLIN3 and CD8, CD4 and CD4/CD8 ratio in situ of OSCC patients. **o** Correlation analysis between *PLIN3* and immune cell infiltrations in HNSCC samples using TIMER. **p** Heatmap illustrating the correlation between PLIN3 and immune checkpoints from cBioportal database. Results are shown by Pearson correlation analysis

Intricate crosstalk between cancer cells and host immune system fuels tumor immune evasion and ultimately results in tumor overgrowth and metastatic dissemination [34]. To explore the immunocytes in situ tissue of PLIN3^+^ HNSCC and OSCC, we utilized cBioPortal database and IHC staining to evaluate the relativity of PLIN3 and CD8, CD4, CD56, CD68, or CD19. In HNSCC, PLIN3 correlated inversely with CD8, CD4, and CD19 densities (Fig. 2a, b, e), while was in direct ratio with CD56 and CD68 (Fig. 2c, d). In OSCC, PLIN3^High^ tumors harbored obviously less infiltrated CD8^+^ T lymphocytes (Fig. 2f, g, l) and CD4^+^ T lymphocytes (Fig. 2h, m), whereas CD56^+^ NK cells, CD68^+^ tumor-associated macrophages (TAMs), and CD19^+^ B lymphocytes were essentially comparable (Fig. 2i–k). Noteworthy, CD4/CD8 ratio < 1 at the tumor–host interface has prognostic value in triple-negative breast cancer [35]; we likewise found the mean CD4/CD8 ratio to be at 0.47, which was less than 1 and negatively associated with PLIN3 (Fig. 2n), indicating that patients with PLIN3^high^ tumor had a poor outcome.

In addition, through the correlation analysis of immune cells markers in HNSCC patients by TIMER database (http://timer.cistrome.org/), we found that patients with high PLIN3 showed increased TGF-β^+^ regulatory T cells and CD68^+^ TAMs, while diminishing CD8^+^ T cells and NOS2^+^ M1 macrophage (Fig. 2o), indicating that PLIN3 favored the establishment of a unique and highly immunosuppressive tumor microenvironment (TME). Furthermore, cBioPortal database showed that PLIN3 enhanced immune checkpoint molecules such as CD244 (2B4), CD274 (PD-L1), interleukin 10 (IL-10), NECTIN2 (CD112), PDCD1LG2 (PD-L2), transforming growth factor beta 1 (TGFβ1), and transforming growth factor beta receptor 1 (TGFβR1) (Fig. 2p).

### OSCC patients with PLIN3^high^ tumor predict the exhaustion of peripheral circulatory T lymphocytes

**Fig. 3:**
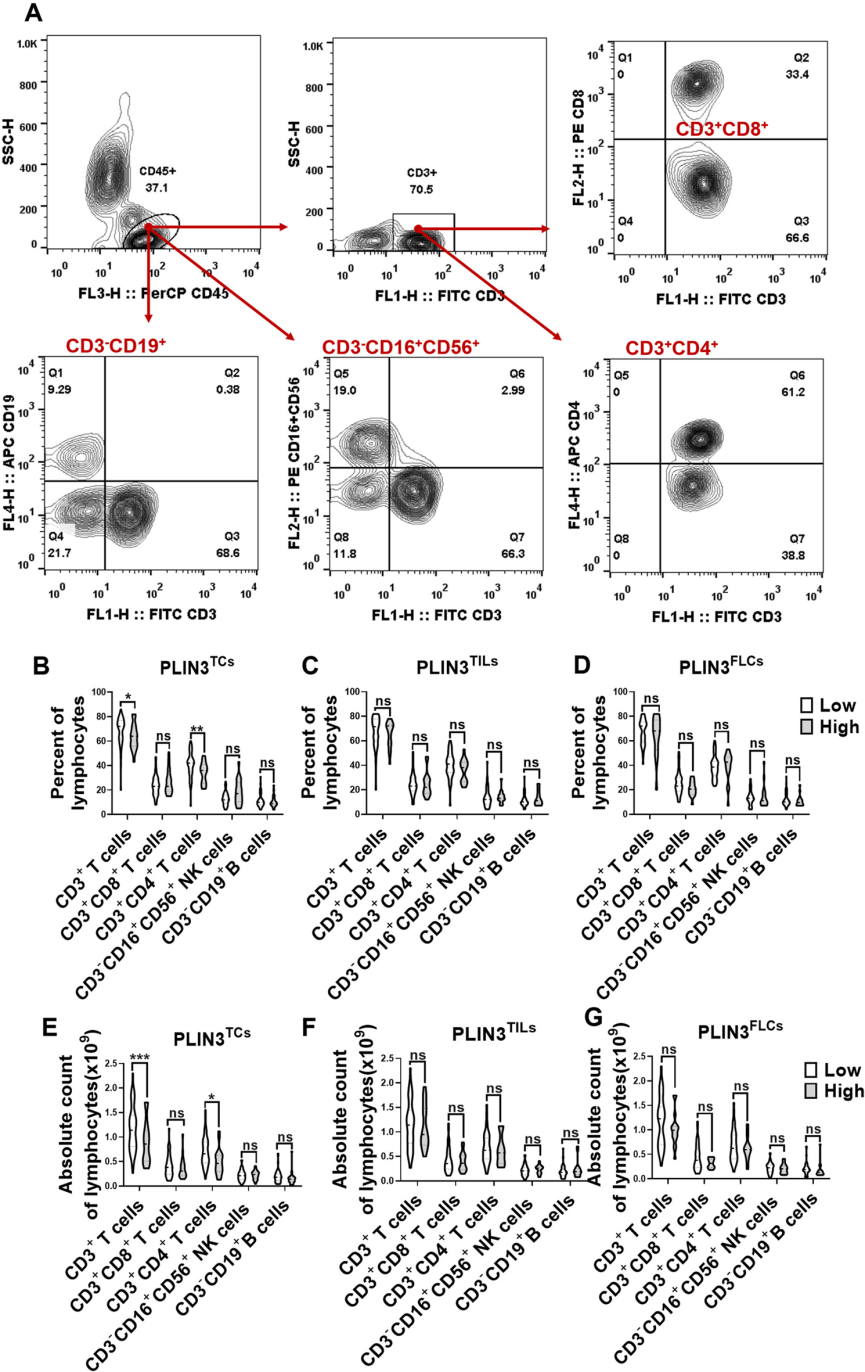
The change of lymphocytes subset in PBMC of OSCC patients according to PLIN3 level. **a** Flow cytometry contour plots showing the strategy for gating lymphocytes. **b–g** The ratio and absolute number of human CD3^+^T cells, CD3^+^CD4^+^ helper/inducer T cells, CD3^+^CD8^+^ cytotoxic T cells, CD3^−^CD16^+^CD56^+^ NK cells and CD3^−^CD19^+^ B cells in blood were analyzed in PLIN3^low^ and PLIN3^high^ groups of TCs, TILs, and FLCs by BD Multitest™ reagent in OSCC patients (n = 74). Results are shown by two-way ANOVA. *p* = Sidak’s multiple comparison test

Since the metastasis of tumor requires cancer cells to circulate in the bloodstream, endure pressure in blood vessels, and escape deadly combat with immune cells [36, 37], we next analyzed the ratio and absolute number of key immunocytes in peripheral blood of OSCC patients (*n* = 74) according to PLIN3 expression by flow cytometry. Human CD3^+^ T cells, CD3^+^CD4^+^ helper/inducer T cells, CD3^+^CD8^+^ cytotoxic T cells, CD3^−^CD19^+^ B cells, and CD3^−^CD16^+^, and/or CD56^+^ NK cells were analyzed in PLIN3^high^ and PLIN3^low^ groups, and the strategy for gating lymphocytes is shown in Fig. 3a. The results indicated that patients with enhanced PLIN3^+^ tumor cells had relatively low ratio and numbers of CD3^+^ T cells and CD3^+^CD4^+^ helper/inducer T cells in blood (Fig. 3b, e), whereas significance was lost within immunocytes (Fig. 3c, f) and fibroblasts (Fig. 3d, g). In addition, the percent and absolute count of NK cells and B lymphocytes were comparable in different cell populations, in agreement with the lack of differences in situ tissue. Patients with PLIN3^high^ tumor cells showed a trend toward lower absolute count of CD8^+^T cell infiltration in TME (Fig. 3e). Collectively, high PLIN3^+^ tumor cells might result in the exhaustion of T cells in peripheral blood.

### Heterogenous PLIN3 in OSCC microenvironment renders distinct clinical prognosis

**Fig. 4:**
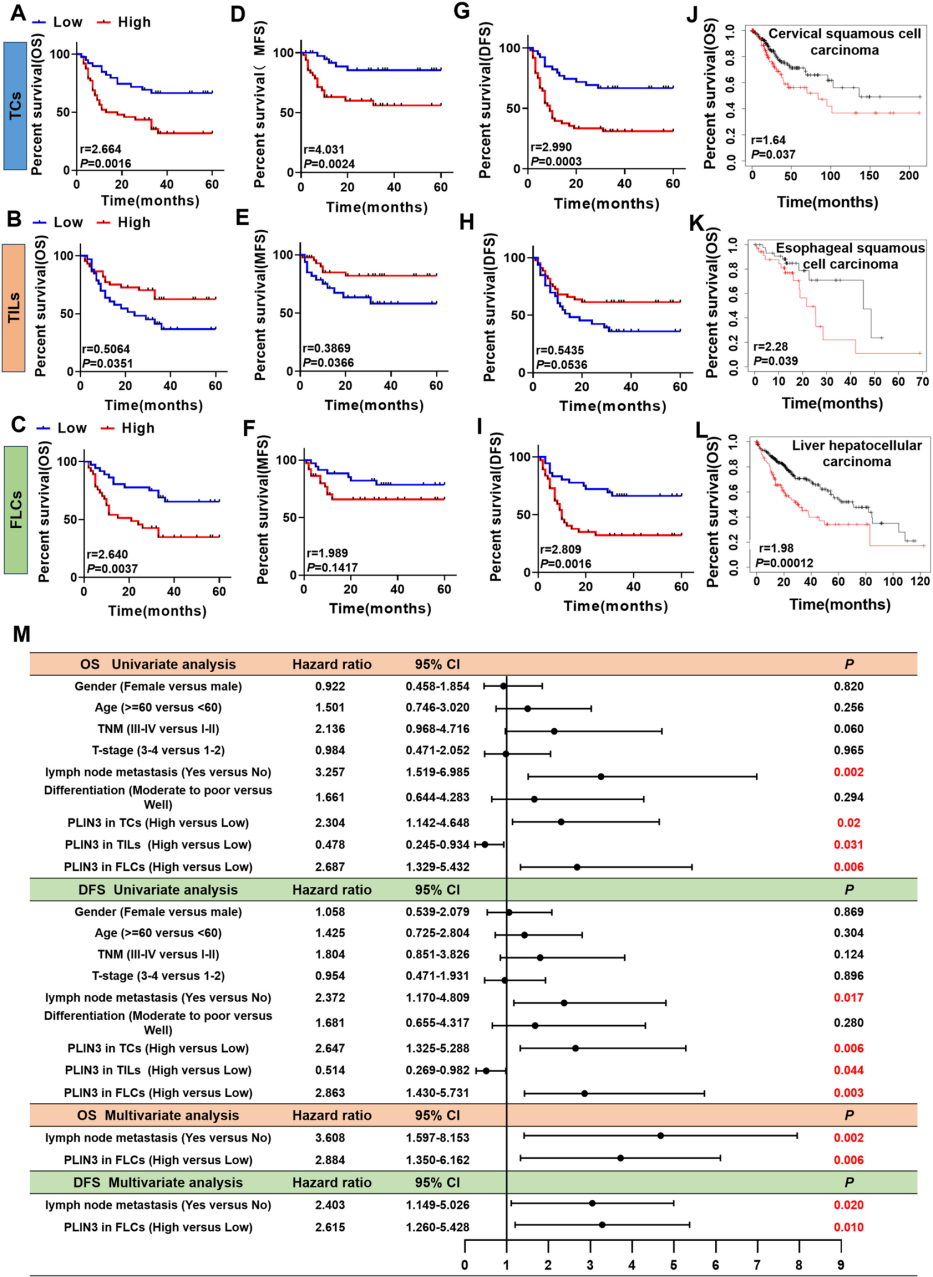
PLIN3 heterogeneity in tumor microenvironment leads to different clinical outcomes. **a–i** Kaplan–Meier survival analyses for overall survival time (OS), metastasis-free survival time (MFS), and disease-free survival time (DFS) of OSCC patients according to the protein expression of PLIN3 in TCs, TILs, and FLCs. **j–l** The effects of PLIN3 expression on the prognosis of OS in cervical squamous cell carcinoma, esophageal squamous cell carcinoma and liver hepatocellular carcinoma patients were shown by Kaplan–Meier plotter database. **m** Cox regression models for OS and DFS in OSCC patients to determine the independent risk factors, adjusted hazard ratio (HR), and 95% confidence interval (CI) of OSCC. Survival analyses including OS, MFS, and DFS were evaluated by Kaplan–Meier and log-rank test

To further clarify the prognostic value of PLIN3, we conducted 5-year survival curve assays. Results showed that patients with PLIN3^high^ tumor cells or fibroblasts had shorter postoperative OS (Fig. 4a, c), MFS (Fig. 4d, f), and DFS (Fig. 4g, i). Conversely, we found that patients with high PLIN3^+^ immunocytes showed longer OS (Fig. 4b), MFS (Fig. 4e), and DFS (Fig. 4h). We hypothesized that high intracellular PLIN3 expression promoted tumor cell growth; however, for immune cells, it enhanced the antitumor immune efficacy of CD8^+^ T lymphocytes. Similar results were observed in cervical squamous cell carcinoma (Fig. 4j), esophageal squamous cell carcinoma (Fig. 4k), and liver hepatocellular carcinoma (Fig. 4l) by Kaplan–Meier plotter database (http://kmplot.com/analysis/index.php?p=service), and patients with enhanced PLIN3 expression in tumor cells had shorter OS.

To analyze the prognostic value of clinicopathological features, we used univariate and multivariate Cox regression analyses. Our data confirmed that the presence of nodal and PLIN3^high^ expression in OSCC was associated with shorter OS and DFS in univariable analysis (Fig. 4m). Moreover, lymph node metastasis (yes vs no, HR 2.403, 95% CI 1.149–5.026, *P* < 0.05, Fig. 4m) and PLIN3 in fibroblasts (high vs low, HR 2.615, 95% CI 1.260–5.428, *P* < 0.05, Fig. 4m) could be used as independent prognostic factors for OS and DFS of OSCC in the multivariable models. Altogether these data indicated that PLIN3 was upregulated in OSCC tissues and high PLIN3^+^ tumor cells may lead to immunosuppression through T cell exhaustion, accompanied by infiltration of PLIN3^+^ fibroblasts, thus collaboratively promoting tumor progression and predicting inferior patient outcome.

### Knockdown of PLIN3 depresses lipid accumulation and the ability of migration in vitro in OSCC

**Fig. 5:**
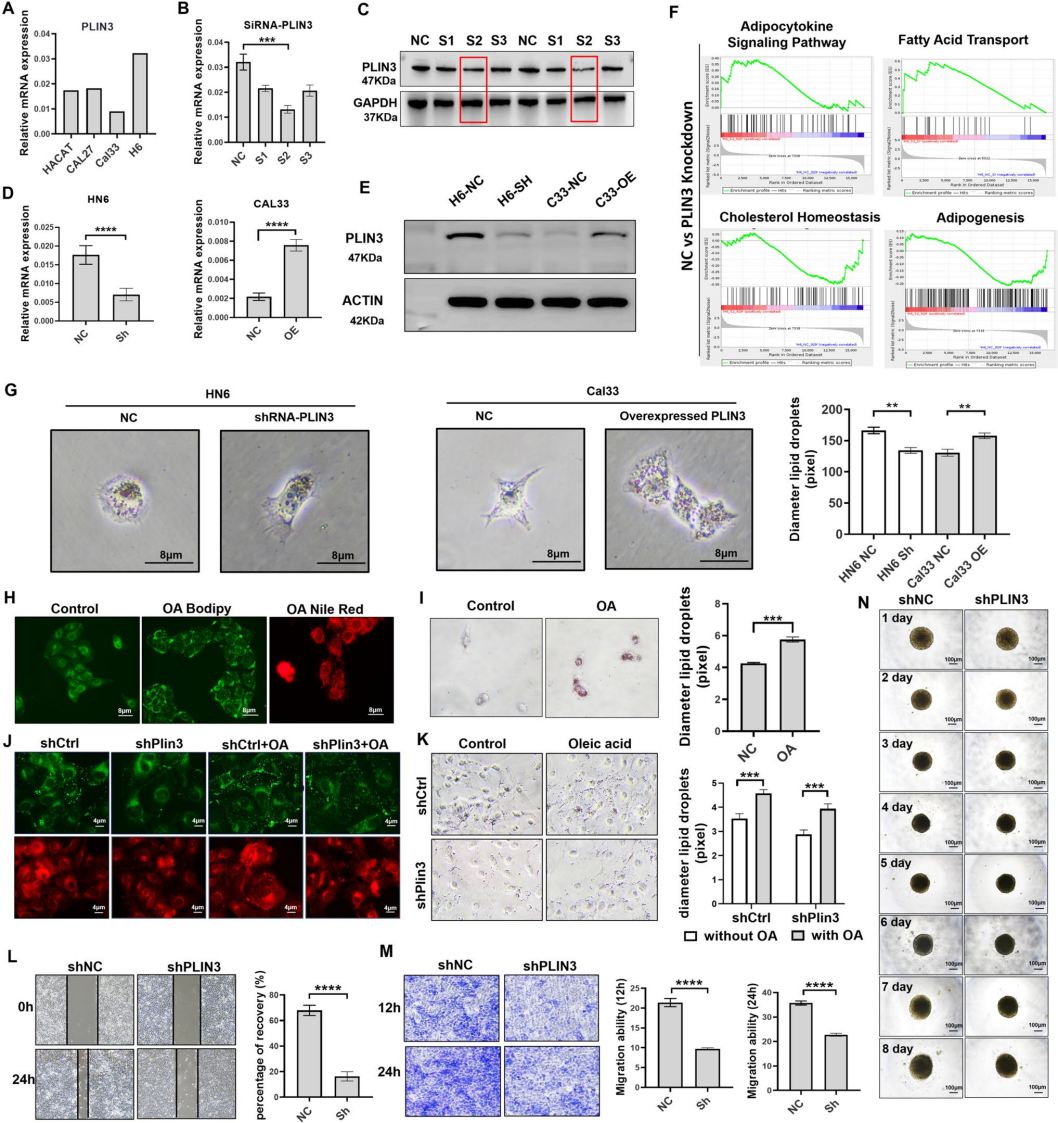
Knockdown of PLIN3 inhibits abnormal lipid accumulation and biological behavior in OSCC cells. **a** PLIN3 expression in human normal keratinocytes (HACAT), Cal27, Cal33, and HN6 cell lines. **b, c** Knockdown expression of PLIN3 in HN6 cells with three independent siRNA, confirmed using qPCR and WB. **d, e** PLIN3-knockdown HN6 or PLIN3-overexpressing Cal 33 cell lines were generated by transfecting lentivirus and validation by qPCR and WB. **f** GSEA of PLIN3 mRNA and OSCC signaling pathways. **g** Oil Red O staining was conducted in HN6 and Cal33 cells. **h–k** Immunofluorescent and Oil Red O were performed to detect LDs content in HN6 cells treated with OA and knocked down with PLIN3. The bar is 8 μm. **l–n** Wound healing and transwell assay; 3D spheroid models were performed to detect HN6 cell migration and proliferation. The results represent the mean ± SEM from three independent experiments. *p* = two-tailed *t* test

Considering the metastasis- and immune-regulatory role of PLIN3 in OSCC patients, we next investigated the role of PLIN3 in OSCC cells in vitro. Firstly, we evaluated the expression level of *PLIN3* in normal epithelial cells and different tumor cells and found it was higher in HN6 and Cal27, but lower in Cal33 (Fig. 5a). We thus knocked down PLIN3 (PLIN3^kd^) in HN6 cell lines with three independent siRNA (Fig. 5b, c) and then established stable PLIN3 silencing HN6 cells and PLIN3 overexpressed (PLIN3^oe^) Cal33 cell lines with lentivirus plasmid vector, and the efficiency was verified by qPCR and WB (Fig. 5d, e).

To discover the potential biological functions and signaling pathways of PLIN3, we obtained KEGG, GO, and HALLMARK annotations in PLIN3^kd^ OSCC cells based on functional enrichment analysis of RNA sequence results. The results showed that PLIN3 played a critical role in multiple lipid metabolism-related pathways including adipocytokine signaling pathway, fatty acid transport, cholesterol homeostasis, and adipogenesis (Fig. 5f). To further investigate whether PLIN3 contribute to LDs biosynthesis, an Oil Red O staining assay was conducted as a visual indicator of intracellular LDs in OSCC. LD deposits were obviously decreased in PLIN3^kd^ HN6 cell lines, but increased in PLIN3^oe^ Cal33 cell lines (Fig. 5g). Consistently, immunofluorescence and Oil Red O staining were performed to identify intracellular vacuole-like structures as being LDs; OA was reported to promote lipid synthesis in HepG2 cancer cells [38] and here also increased the extent of LDs in OSCC cells (Fig. 5H, I). Moreover, the intracellular LDs were smaller and less abundant in the PLIN3-knockdown HN6 cells than in the controls, but the addition of exogenous OA restored LD biogenesis (Fig. 5j, k). Multiple pieces of evidence demonstrated LDs were responsible for incusing cancer cells proliferation and migration [39]. As expected, knocking-down PLIN3 led to a significant reduction in the migration of HN6 cells in wound healing and transwell assays (Fig. 5l, m), but failed to influence proliferation when cells were cultured continuously for 8 days by 3D spheroid models (Fig. 5n).

### PLIN3^high^ tumor cells maintain high B7-H2 level and induce CD8^+^T cells exhaustion in OSCC patients

**Fig. 6:**
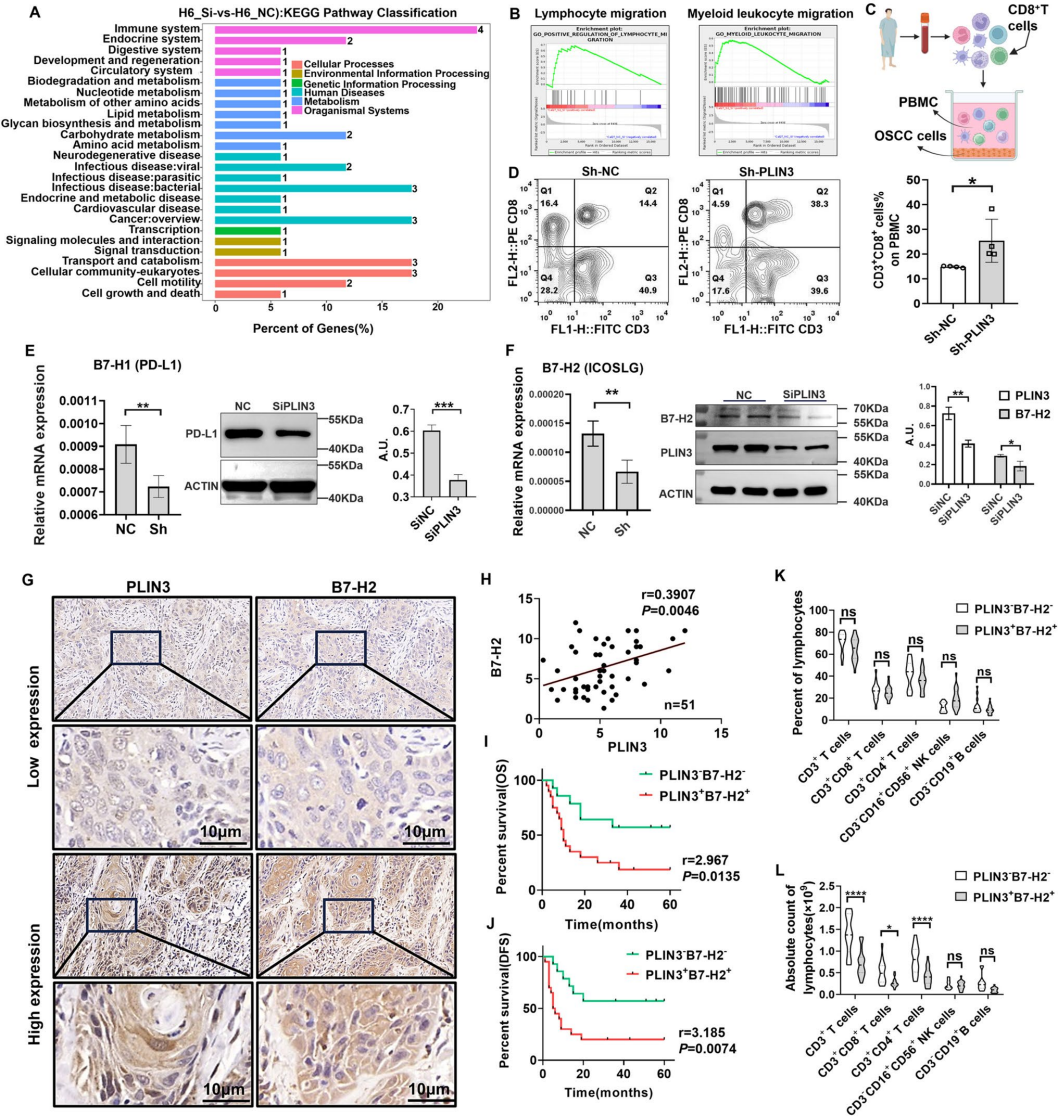
PLIN3 upregulates B7-H2/PD-L1 level and induces CD8^+^T cells exhaustion in OSCC patients. **a** KEGG pathway classification of PLIN3 in OSCC cells. **b** GO annotated positive regulation of lymphocyte migration and myeloid leukocyte migration. **c** Schematic diagram of human tumor cells/immunocytes co-culture system. **d** After co-cultured with PLIN3 knockdown HN6, the percentage of CD3^+^ CD8^+^ T cells in PBMC were analyzed. **e**, **f** qPCR and WB analysis showed the mRNA and protein expression of PD-L1 and B7-H2 in HN6 cells. **g**, **h** Correlation between PLIN3 and B7-H2 shown by immunohistochemistry and quantitative analysis (*n* = 51). **i**, **j** Kaplan–Meier survival curves for overall survival time (OS) and disease-free survival time (DFS) of OSCC patients according to the expression of PLIN3 and B7-H2 in tumor cells. **k, l** The ratio and absolute count of lymphocytes subset of PBMCs in OSCC patients with distinct PLIN3 and B7-H2 expression in tumor cells

LDs are linked to the regulation of immune by acting as specialized hubs of signaling with lipid mediator formation in cancer [26, 39]. Consistently, differential changed genes by PLIN3 knockdown were significantly enriched in pathways of immune system (Fig. 6a), and enrichment plot revealed that PLIN3 could enrich the migration of lymphocytes and myeloid leukocytes (Fig. 6b). To further estimate the role of PLIIN3^+^ tumor cells in immune escape, we established human tumor/immunocytes co-culture system using peripheral blood mononuclear cells (PBMCs) (Fig. 6c) and performed flow cytometry to detect CD8^+^ T cell activation ratio. The results showed that the proportion of CD3^+^CD8^+^ T cells was significantly upregulated in PLIN3^kd^ HN6 cells compared to controls (Fig. 6d). As reported previously, increased LDs associated with reduced recruitment of IFNγ-secreting CD8^+^ T cells to tumor site could consequently avoid exposure of PD-L1 and PD-1 on tumor and CD8^+^ T cells [40]. We indeed observed a significant downregulation of B7-H1 (PD-L1) and B7-H2 (ICOSLG) mRNA and protein expression in PLIN3^kd^ HN6 cells (Fig. 6e, f). Patients with B7-H2^high^ tumor cells showed advanced TNM stage and N^+^ stage with decreased OS, MFS, or DFS, and this sub-cohort was featured with a trend of diminished CD4^+^ T cells and CD8^+^ cells in tumor center in situ, which positively correlated with PD-L1 and IL10 [41]. In this study, we focused on PLIN3/B7-H2 signals in OSCC and correlation between PLIN3 and PD-L1 was investigated in our other unpublished research.

**Fig. 7:**
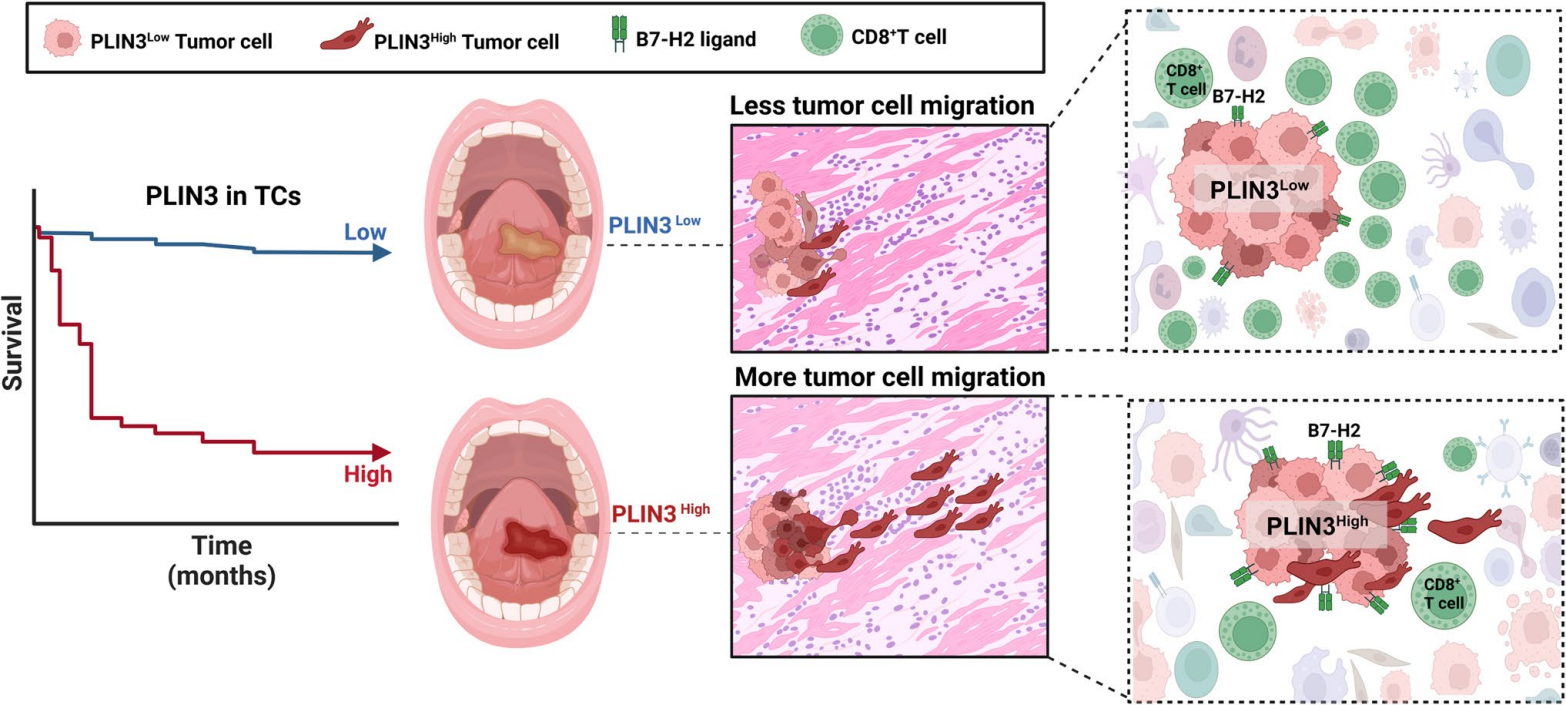
Schematic of the study. Patients with PLIN3^high^ tumors had a poor prognosis and displayed a more likely to occur distant metastasis, which exhibited as high intensity B7-H2 expression and less infiltrated CD8^+^ T cells. *p < 0.05, **p < 0.01, ***p < 0.001, ****p < 0.0001, ns, no significance

Given that PLIN3 promoted B7-H2 protein expression, we reasoned that B7-H2 was required for PLIN3-mediated tumor promotion. To test our hypothesis, we analyzed the expression of PLIN3 and B7-H2 in the same tissue sections of OSCC by IHC staining (*n* = 51), and the results showed that the protein levels of B7-H2 were largely upregulated in PLIN3^High^ tumors (*n* = 51), which was positively correlated (Fig. 6g, h). Thus, we divided OSCC patients into different subpopulations based on PLIN3 and B7-H2 expression, and OSCC patients with high PLIN3^+^B7-H2^+^ tumor cells had significantly shorter OS and DFS (Fig. 6i, j) and harbored less absolute count of CD3^+^CD8^+^ and CD3^+^CD4^+^ T cell infiltration in peripheral blood (Fig. 6k, l). Compelling evidence suggests that LDs accumulation led to failure in dendritic cells (DCs) maturation, which limited recruitment/activation of naïve CD8^+^ T cells via co-stimulatory factors CD80/86/MHC-I and CD28/TCR [40]. These data indicated that LDs-related PLIN3 promoted CD8^+^T cells exhaustion in OSCC patients, which might be related to B7-H2-mediated immune homeostasis (Fig. 7).

## Discussion

PLIN3, as a surface protein of LDs, can regulate LD biosynthesis and degradation [42]; PLIN3 associates with LDs in a manner that is responsive to the status of neutral lipid synthesis and storage in the cell [21]. At the same time, PLIN3 plays an important role in cell viability and autophagy, which can lead to drug resistance to treatment [43]. Kaplan–Meier curves revealed that elevated PLIN3 predicted poor DFS and OS in clear cell renal cell carcinoma [22]. In prostate cancer, PLIN3 overexpression promotes tumor progression [43]. Similar to previous studies, we demonstrated that PLIN3 was upregulated and predicted a poor prognosis in OSCC, suggesting that PLIN3 accumulated in cells to serve as a conservative oncogene to promote cancer development.

Trempolec et.al reported the secretion of active TGF-β2 drives a significant increase in LD amounts within DCs and alters their metabolic status in such a way that it contributes to impair DC activity, including through a defect in antigen presentation and T cell activation [25]. We found that PLIN3 was inversely associated with CD4/CD8 in tumor tissue and knocking-down PLIN3 can reduce intracellular LDs deposits, which was consistent with the previous findings [42], making us turn to investigate immune checkpoint molecules. B7-H2 as B7 homolog binds not only to the inducible co-stimulator (ICOS) but also to the CD28 [44], and the functional study of B7-H2 found that it can promote the production of IL-4 and IL-10, thereby inhibiting the activity of CD8^+^ T lymphocytes in TME [45]. Consistently, our research showed that PLIN3 upregulated the expression of PD-L1 and B7-H2 (ICOSLG). Previous studies also supported the role of B7-H2 in tumor progression: Patients with B7-H2^high^ tumors showed high TNM stage and lymph node metastasis with less infiltrated CD8^+^ cells in OSCC tumor center and in blood and were connected to worse survival (OS, DFS) in OSCC patients [41], which was consistently observed in our study. In summary, PLIN3 induced apoptosis of CD8^+^ T lymphocytes and promoting the expression of PD-L1 and B7-H2 in OSCC.

In conclusions, we uncovered the tumorigenic-promoting function of PLIN3 in OSCC. Elevated PLIN3 expression in patients correlated with shorter survival time and postoperative distant metastasis. Furthermore, PLIN3 inhibited CD8^+^ T cells infiltration not only in the tumor microenvironment but also in circulating lymphocytes, which might be regulated by B7-H2.

## Declarations

### Availability of data and material

The datasets generated during and/or analyzed during the current study are available from the corresponding author on reasonable request.
